# Editorial: Neural Plasticity and Behavioral Responses to Adversity

**DOI:** 10.3389/fnbeh.2022.969371

**Published:** 2022-07-01

**Authors:** Nuno Dinis Alves

**Affiliations:** ^1^Department of Psychiatry, Columbia University, New York, NY, United States; ^2^New York State Psychiatric Institute, New York, NY, United States

**Keywords:** neural plasticity, adversity, homeostasis, behavior, adaptation

The mammalian brain has a remarkable capacity to overcome internal (e.g., genetic susceptibility) and external insults (e.g., stress) that may perturb its homeostasis by maintaining physiological functions at optimal levels and preventing allosteric load, a serious physical and mental health threat. This intrinsic property is observed in many brain regions including limbic and cortical areas. For example, the hippocampus and the prefrontal cortex (PFC) show various forms of neural plasticity, including the remodeling of neuronal cytoarchitecture, glial and synaptic plasticity, (epi)genetic modifications and, specifically in the hippocampus, the postnatal generation of new neural cells (cytogenesis). Throughout life, and particularly during developmental and sensitive periods, these neurobiological processes respond to varying adverse or enriched environmental changes leading to adaptive or maladaptive changes. Depending on factors such as timing, severity and, duration of experiences or insults, such neuroplastic events are thought to play a crucial role, being involved in either a beneficial influence, coping with subsequent events, or detrimental, impairing behavior and ultimately raising the susceptibility to develop mental illness. Despite increased attention to these processes, is still to be fully uncovered the involvement of neural plastic mechanisms in the susceptibility and resilience to adversity and, its therapeutic contribution to rescuing behavioral impairments.

This Research Topic explores the changes and actions evoked by neural plastic mechanisms in response to environmental changes, particularly during early life. Ventura et al. highlight the influence of interactions between genotype, sex, and previous stress experiences on the development of individual (mal)adaptive coping strategies. Also, the authors point out a central role of the meso-cortico-limbic dopaminergic system in the expression of coping strategies. In the context of early-life adversity (ELA), Milbocker et al. review the impact of different forms of adversity (distinct in timing, predictability and intensity) on critical neurodevelopmental periods (perinatal and adolescence) and their long-term maladaptive (or adaptive) behaviors. In particular, the authors focus on the alterations promoted by intense and unpredictable ELA on normal proliferation and function of glial cell types (astrocytes, microglia and oligodendrocytes) in the hippocampus and PFC that ultimately impair synaptic pruning and myelination, crucial processes for proper brain circuit refinement and intact related behaviors. Additionally, the authors review glia-centric interventions with the potential to mitigate the ELA-induced detrimental changes in behavior. One of these therapeutic strategies is parental care. Infants' sensitivity and response to changes in parental care are explored by Graf et al.. The authors review the neuroplastic mechanisms underlying the critical contribution of caregiver sensory stimuli (e.g., visual, auditory, olfactory and, tactile) to regulate infant's brain functions, as well as physiological and emotional homeostasis, and explore the bidirectional brain response to nurturing maternal care and maltreatment. At a morphological and structural level, Poleksic et al. studied the consequences of acute early-life stress in the adult PFC. Using a maternal deprivation (MD) model [24 h isolation at postnatal day (PND) 9] in male rats, Poleksic et al. revealed a long-term region-specific decrease in the density of interneurons [glutamate decarboxylase (GAD)67^+^] and subtypes parvalbumin (PV)^+^ and cholecystokinin (CCK)^+^, increase in brain-derived neurotrophic factor (BDNF) expression and an area/layer-specific reduction in microglial activation denoted by a decrease in Iba1^+^ cell density. Interestingly, cellular and molecular changes induced by MD were accompanied by a mild impairment of cognitive flexibility, a behavior task typically associated with intact PFC function. The authors suggest that upregulation of PFC BDNF, important for interneuron density, constitutes an adaptative and compensatory response to the MD-induced reduction of gamma-aminobutyric acid (GABA)ergic cell density, which consequently hampers the impact of MD on cognitive flexibility. To study the impact of modulating early life environmental changes on pro-social behavior, Kalamari et al. developed an operant liberation task to measure the motivation to help a trapped (and not trapped) conspecific in adult rats. Specifically, the authors promoted negative (MD on PND3) and positive (enriched complex housing from PND26 onwards) early life changes and measured the motivation for pro-social behavior in adulthood. In this original article, the authors observed a slight and non-significant reduction in motivation for prosocial activity promoted by MD, yet surprisingly complex housing led to a reduced motivation to gain access to a trapped (and non-trapped) cage mate, even when liberation does not promote social contact. These complex housing-induced decrease in the motivation was social reward-specific as no alterations were observed in motivation to work for a sucrose reward. Of notice, increased levels of distress denoted by the number of ultrasonic vocalizations were correlated with decreased motivation to liberate the cage mate, suggesting a reduced active helping behavior than when animals are less stressed. They hypothesize that these observations might be linked to a decreased appeal for social activity in complex housing rats in comparison to those housed in standard conditions.

Finally, in a multiscale perspective, Kawatake-Kuno et al. summarize the neural plastic mechanisms underlying the peculiar fast and sustained antidepressant action of the N-methyl-D-aspartate (NMDA) antagonist ketamine, particularly in the hippocampus and PFC. The authors discuss the involvement of glutamatergic neurotransmission in ketamine's fast therapeutic actions and refer to the Ca^2+^-signaling-mediated rescue of neuronal processes (e.g., dendritic alterations, synaptic plasticity and, epigenetic modifications) that contribute to its long-lasting effects.

The review articles and novel data reported in original studies of this Research Topic add to the knowledge regarding the structural and behavioral impact of environmental changes throughout life, and further contribute to highlighting the importance of different mechanisms of neural plasticity to (mal)adaptive coping strategies against adversity.

## Author Contributions

NA defined the conception and wrote the manuscript.

## Conflict of Interest

The author declares that the research was conducted in the absence of any commercial or financial relationships that could be construed as a potential conflict of interest.

## Publisher's Note

All claims expressed in this article are solely those of the authors and do not necessarily represent those of their affiliated organizations, or those of the publisher, the editors and the reviewers. Any product that may be evaluated in this article, or claim that may be made by its manufacturer, is not guaranteed or endorsed by the publisher.

